# Benzyl isothiocyanate exhibits antibacterial and antibiofilm activity against *Fusobacterium nucleatum* while preserving viability of human bone marrow mesenchymal stem cells: an *in vitro* study

**DOI:** 10.3389/fcimb.2025.1683203

**Published:** 2025-10-24

**Authors:** Abdulaziz A. Almobarak, Abdullah Alqedairi, Abdulaziz Binrayes, Rhodanne A. Lambarte, Abdurahman A. Niazy, Fahd Aljarbou

**Affiliations:** ^1^ Department of Restorative Dental Sciences, Endodontic Division, College of Dentistry, King Saud University, Riyadh, Saudi Arabia; ^2^ Department of Prosthetic Dental Sciences, College of Dentistry, King Saud University, Riyadh, Saudi Arabia; ^3^ Molecular and Cell Biology Laboratory, Prince Naif bin Abdulaziz Health Research Center, King Saud University Medical City, Riyadh, Saudi Arabia; ^4^ Department of Oral Medicine and Diagnostic Sciences, College of Dentistry, King Saud University, Riyadh, Saudi Arabia

**Keywords:** benzyl isothiocyanate, *Fusobacterium nucleatum*, antimicrobial, antibiofilm, calcium hydroxide, regenerative endodontics, stem cells, viability

## Abstract

**Background:**

Regenerative endodontic procedures require effective disinfection and preservation of stem cell viability, which are essential for successful tissue regeneration. Conventional intracanal medicaments possess antibacterial properties but may negatively impact stem cell survival. Benzyl isothiocyanate (BITC) is a bioactive compound with known antibacterial properties and favorable biocompatibility.

**Methods:**

This study evaluated the antibacterial efficacy of BITC against *Fusobacterium nucleatum* in both planktonic and biofilm forms and assessed its biocompatibility on human bone marrow mesenchymal stem cells (hBMMSCs) in comparison to calcium hydroxide (Ca(OH)_2_). Minimum inhibitory concentration (MIC) and minimum bactericidal concentration (MBC) were determined using microdilution and agar culture methods. Biofilm biomass was quantified using the crystal violet assay, viability was visualized through live/dead imaging, and microstructure was examined using scanning electron microscopy. Stem cell viability was evaluated using the Alamar Blue™ assay, and cell morphology was examined using inverted microscopy.

**Results:**

BITC exhibited MIC and MBC values of 0.2% and 0.4%, respectively. At 0.2%, BITC demonstrated comparable antibacterial activity to Ca(OH)_2_, whereas 0.4% and 0.8% showed enhanced bactericidal effects. Notably, 0.2% and 0.4% BITC maintained hBMMSCs viability and morphology, while 0.8% BITC and Ca(OH)_2_ significantly reduced cell survival.

**Conclusion:**

These findings suggest that BITC could serve as a potential antimicrobial agent in regenerative endodontic treatment and future therapeutic applications.

## 1 Introduction

Regenerative endodontic procedures (REPs) were first introduced by Banchs and Trope in 2004 to treat necrotic permanent immature teeth (Banchs and Trope, 2004). The primary goal of REPs is to eliminate symptoms, promote apical bone healing, continue root development, and close the root apex (Kim et al., 2018). To achieve these goals, three important biological factors must be present: stem cells, growth factors, and scaffolds (Hargreaves et al., 2013). Recently, the role of disinfection has received more attention in regard to the success of regeneration, with an emphasis on additional detoxification that clears infections and maintains stem cell viability through intracanal medicaments (Diogenes and Hargreaves, 2017). The most commonly used intracanal medicaments in REPs are antibiotic pastes and calcium hydroxide (Ca(OH)_2_) (Kontakiotis et al., 2015). Several studies have reported successful outcomes after using various antibiotic mixtures in REPs (Chan et al., 2017; Lin et al., 2017). However, potential side effects such as tooth discoloration, the development of antibiotic resistance, and negative effects on stem cell proliferation have been major concerns (Sedgley et al., 2008; Torabinejad et al., 2017).

As a result, Ca(OH)_2_ has been widely favored for its proven antimicrobial properties and successful regenerative outcomes (Desai and Chandler, 2009; Glynis et al., 2021). These properties are primarily due to its high alkalinity and the ability to release hydroxyl ions, which create an unfavorable environment for bacterial growth (Mohammadi and Dummer, 2011). However, the antimicrobial effect of Ca(OH)_2_ remains limited in some cases, particularly in persistent infections and against certain resistant bacteria such as *Fusobacterium nucleatum* (*F. nucleatum*) (Sathorn et al., 2007; Ferreira et al., 2015). This limitation is critical in REPs, since *F. nucleatum* is frequently detected in necrotic immature permanent teeth and endodontic abscesses and often persists after instrumentation and chemical disinfection (Zandi et al., 2018; Fouad, 2019). In addition to the microbial limitation of Ca(OH)_2_, its effect on host stem cells remains controversial. While some studies reported that Ca(OH)_2_ promotes the proliferation and osteogenic differentiation of dental pulp stem cells (DPSCs) in certain conditions (Chen et al., 2016), other studies suggest unfavorable effects on DPSCs proliferation (Alghilan et al., 2017). Given these limitations and conflicting outcomes, the need to develop an antimicrobial agent that can effectively eradicate bacterial infections while maintaining stem cell viability, promoting cell proliferation, and ultimately enhancing the outcome of REPs remains important in regenerative endodontic research.

Natural products have been used in medicine for centuries due to their relatively minimal side effects and notable antimicrobial properties (Garg et al., 2014). Benzyl isothiocyanate (BITC) is a naturally occurring bioactive compound found in various plants, including *Salvadora persica, Carica papaya*, and *Tropaeolum majus* (Sofrata et al., 2011; Kaiser et al., 2017; Zhang and Chen, 2017). This metabolite is synthesized by plants in reaction to disruption, mainly via glucosinolate and myrosinase hydrolysis (Romeo et al., 2018b). BITC has been recognized for its significant antibacterial effects against a wide range of gram-positive and gram-negative bacterial pathogens. It has been shown to downregulate virulence gene expression in *Staphylococcus aureus* and inhibit *Salmonella typhimurium* motility and biofilm growth (Wang et al., 2019; Niu et al., 2020). It was also reported to have an antimicrobial efficacy comparable to that of gentamycin sulfate in treating *Pseudomonas aeruginosa* infections, with minimal toxicity and lower inflammatory responses (Yang et al., 2024). Additionally, extracts containing BITC have been shown to inhibit oral biofilm formation, reduce periodontal pathogens, and promote gingival wound healing (Aljarbou et al., 2022; Harun et al., 2023). These antibacterial properties are attributed to the ability of BITC to protrude bacterial membranes, disrupt metabolic activity, and induce oxidative stress, ultimately leading to cell death (Hoch et al., 2024). However, the antibacterial effects of BITC against *F. nucleatum* have yet to be explored.

In cancer research, BITC has been studied for its anticancer properties, exhibiting a promising capacity to induce apoptosis, cell cycle arrest, and oxidative stress in cancer cells (Dinh et al., 2021). BITC downregulates Mcl-1, an anti-apoptotic protein, in leukemia cells, triggering apoptosis via caspase activation. In contrast, it exhibits minimal toxic effects on normal human peripheral blood mononuclear cells (Zhou et al., 2013). Furthermore, BITC has demonstrated cytotoxic effects against canine lymphoma and leukemia cells by inducing caspase-dependent apoptosis through the upregulation of reactive oxygen species (ROS), causing DNA damage (Henklewska et al., 2021).

Despite extensive research on the anticancer effects of BITC, its effects on normal mesenchymal stem cells remain poorly understood. Several studies on moringin, in which BITC is the main bioactive compound, have demonstrated that it has positive effects on cell survival and proliferation of human periodontal ligament stem cells (hPDLSCs) and human gingival mesenchymal stem cells (hGMSCs) (Romeo et al., 2018a; Chiricosta et al., 2019). However, the effects of BITC on the viability of human bone marrow stem cells (hBMMSCs) have not been investigated. Therefore, the present study aimed to evaluate the antibacterial properties of BITC against *F. nucleatum* in both planktonic and biofilm states and to assess its effect on the viability of hBMMSCs in comparison to Ca(OH)_2_.

## 2 Materials and methods

### 2.1 Bacterial strain and culture preparation

Before each experiment, *F. nucleatum* ATCC 25586 (Cat. No. 0328K, Microbiologics KWIK-STIK™, Saint Cloud, MN, USA) was cultured on tryptic soy agar (TSA) plates supplemented with hemin and vitamin K (Watin-Biolife Factory, Riyadh, Saudi Arabia) under anaerobic conditions at 37 °C for 72 h using an anaerobic jar and generator pack (AnaeroPack™, Mitsubishi Gas Chemical Co., Tokyo, Japan). Single isolated bacterial colonies were then inoculated in 10 mL tryptic soy broth (TSB) (Watin-Biolife Factory) supplemented with 5 µg/mL hemin and 1 µg/mL menadione and incubated anaerobically in an Excella E24 incubator shaker (New Brunswick Scientific, Edison, NJ, USA) for 24 h. After incubation, the bacterial concentrations were standardized to an optical density (OD600) of approximately 0.5-0.6 (mid-log phase) using a spectrophotometer (Libra S22, Biochrom Ltd., Cambridge, UK) before subsequent experiments were performed, unless otherwise noted (Casasanta et al., 2020).

### 2.2 Chemicals and experimental groups

BITC (98% purity) in liquid form was obtained from Sigma-Aldrich (Cat. No. 252492, St. Louis, MO, USA) and freshly dissolved in dimethyl sulfoxide (DMSO, 0.2%, Sigma-Aldrich) immediately prior to the experiments. Based on preliminary evaluation, the following concentrations were selected: 0.05%, 0.1%, 0.2%, 0.4%, 0.8%, and 1.6%. As a positive control group, calcium hydroxide was purchased in the form of UltraCal™ XS (35% Ca(OH)_2_, Ultradent, South Jordan, UT, USA).

### 2.3 Determination of minimum inhibitory concentration and minimum bactericidal concentration

To determine the MIC, a modified resazurin microplate-based assay method was used (Franzblau et al., 1998). Briefly, a BITC stock solution was prepared in DMSO and serially diluted using TSB to obtain the concentration range. A sterile 96-well U-bottom microtiter plate was prepared by adding 150 μL of 1.6% BITC into Column 8, and 150 μL of TSB to Columns 3-7, followed by two-fold serial dilutions using a multi-channel pipette to generate descending concentrations ranging from 1.6% to 0.05%. Ca(OH)_2_ and DMSO served as positive controls, TSB with a bacterial inoculate as a bacteria growth control, and TSB alone served as a negative control. Fifty microliters of the standardized *F. nucleatum* suspension were added to the appropriate wells, reaching the final working volume to 200 μL. Plates were incubated anaerobically at 37 °C for 24 h. After the incubation period, 20 μL 0.015% resazurin indicator solution (Sigma-Aldrich) was added to all wells and incubated for 2 h. A color change from blue to pink indicates metabolic activity, whereas no color change indicates bacterial growth inhibition. The MIC was determined as the lowest concentration at which no change in color was observed (Dias et al., 2014; Elshikh et al., 2016).

For MBC determination, 20 µL aliquots from the MIC-related wells in the 96-well plates were cultured on Brucella blood agar plates supplemented with hemin and vitamin K (Watin-Biolife Factory, Riyadh, Saudi Arabia) and incubated overnight at 37 °C under anaerobic conditions. The MBC was identified as the lowest concentration with no visible bacterial colony growth (Sabrah et al., 2013). All experiments were conducted in triplicates from three independent biological replicates.

### 2.4 pH measurements

The pH of the experimental solutions was measured using a calibrated pH meter (Jenway 3540 pH & Conductivity meter, Bibby Scientific, Staffordshire, UK). Calibration was performed before each reading using standard buffer solutions at pH 4.0, 7.0, and 9.0 (Honeywell Fluka™, Seelze, Germany). Samples were prepared in 15 mL conical tubes (TPP^®^, Trasadingen, Switzerland), each containing 10 mL of supplemented tryptic soy broth. All tubes were incubated at 37 °C, and pH measurements were recorded at 1, 3, and 7 days.

### 2.5 Planktonic bacterial viability assessment

Following MIC determination, the same experimental setup was used to evaluate the viability of planktonic *F. nucleatum*. The fluorescence readings (FI) were recorded on days 1, 3, and 7 using a Synergy HT microplate reader (BioTek Instruments, Winooski, VT, USA) at excitation and emission wavelengths of 530 and 590 nm, respectively (Niazy et al., 2024). The percentage of bacterial viability was calculated using the following equation:


F. nucleatum cell viability % = (mean FI of sample/mean FI of bacterial control) × 100

### 2.6 Reactive oxygen species production

The intracellular production of reactive oxygen species (ROS) in *F. nucleatum* following BITC treatment was assessed using a fluorescence-based assay (Nowicki et al., 2016). Bacteria were cultured in TSB to an OD_600_ = 0.5, then harvested and washed three times with phosphate-buffered saline (PBS). The fluorogenic probe 2',7'-dichlorofluorescein diacetate (DCFH-DA; Santa Cruz Biotechnology, Heidelberg, Germany) was added to a final concentration of 30 μM, and the cells were incubated anaerobically at 37 °C for 60 min in the dark with shaking. After incubation, cells were centrifuged and washed twice with PBS and resuspended in fresh TSB. Bacterial suspensions were then treated with different BITC concentrations for 6 h at 37 °C under anaerobic conditions. For controls, untreated samples and groups treated with DMSO or Ca(OH)_2_ were included under the same conditions. Fluorescence intensity was measured using a Synergy HT microplate reader (BioTek Instruments, Winooski, VT, USA) at an excitation wavelength of 488 nm and emission wavelength of 535 nm. ROS levels were expressed as relative fluorescence units (RFU).

### 2.7 Biofilm biomass quantification

To quantify biofilm formation, a crystal violet biofilm assay was used as described by haney et al (Haney et al., 2018). Briefly, BITC concentrations and control groups were prepared as previously described. After bacterial culturing was carried out, each well of the 96-well plate was inoculated with 150 μL of bacterial suspension at an OD of 0.5 at 600 nm. Untreated bacterial growth controls served as negative controls, and the experiment was conducted at two time points: days 3 and 7. At each time point, non-adherent bacteria were removed by gently washing the 96-well plates thrice using distilled water. The plates were left to air-dry for 20 mins, and then 150 μL of 0.5% crystal violet solution (Sigma-Aldrich) was added to each well. After staining, the wells were washed with distilled water to remove excess dye and dried at 37°C for 15 min. Absolute ethanol was added to each well, and the wells were incubated for 15 mins. Then, the absorbances were read spectrophotometrically at 570 nm using a BioTek Synergy HT microplate reader. Biofilm inhibition was calculated as a percentage relative to the untreated bacterial control. The experiments were all performed in triplicate.

### 2.8 Biofilm viability assessment

The effects of experimental groups on *F. nucleatum* biofilms were further assessed using live/dead fluorescent imaging and viewed under confocal laser scanning microscopy (CLSM). Bacterial cells were inoculated into 2 mL of supplemented TSB at (OD_600_ = 0.5) initial turbidity and cultured on coverslips in 6-well culture plates at 37°C in an anaerobic chamber for 3 and 7 days. BITC stock solutions and control groups were prepared as previously described. For the live/dead staining of biofilms, any remaining broth was removed from each well, and the coverslips were washed gently three times with PBS and transferred to a new 6-well plate. About 200 µL of working solution (LIVE/DEAD^®^ BacLight Bacterial Viability kit, Invitrogen, Eugene, OR, USA) was added to each biofilm sample, following the manufacturer's instructions, and then incubated for 30 min at room temperature, protected from light. The stained surface was placed face down onto a clean microscope slide and imaged using a Nikon C2 CLSM (Nikon Instruments Inc., Tokyo, Japan) with a 20×/0.75 NA air objective using (λexc) 488 nm/(λem)<550 nm for SYTO^®^ 9 (BacLight™ Component A) and (λexc) 568 nm/(λem) >600 nm for propidium iodide (BacLight™ Component B). Image analysis was carried out using ImageJ software (version 1.50i, NIH, Bethesda, USA) (Niazy et al., 2024). The green fluorescence represents live cells, and red represents dead cells. The percentages of bacterial cell death were calculated as previously described using the following equation.


$$\begin{array}{c} F.\;nucleatum\text{ cell death }\%\;\\ =\text{ red fluorescence }\left(\text{dead bacteria}\right)/\text{total fluorescence }\left(\text{live }+\text{ dead bacteria}\right)\;\times \;100 \end{array}$$

### 2.9 Bacterial morphology analysis

Bacterial samples were grown for 48 h and then cultured for 7 days, as described above. The culture broth in each well was removed, and the cover slips were washed with PBS. The scanning electron microscopy (SEM) sample preparation method was conducted as previously described by Diarra et al (Diarra et al., 2023). Cover slip samples were fixed with 2.5% glutaraldehyde solution at 4°C for 2 h, washed with PBS three times, and dehydrated serially with increasing concentrations of ethanol (30 to 95%) for 10 mins each, followed by two 15 min washes with absolute ethanol. Finally, each coverslip was critically point-dried and coated with gold using a JEOL JFC1100 sputter instrument (JEOL Ltd., Tokyo, Japan) and then viewed using a JEOL JSM-6360LV scanning electron microscope (JEOL Ltd., Tokyo, Japan).

### 2.10 Cell culture

Immortalized human bone marrow-derived mesenchymal stem cells (hMSC-TERT20) were used as an experimental model for *in vitro* biocompatibility studies (Liu et al., 2015). Immortalization with human telomerase reverse transcriptase was performed for the mesenchymal stem cells following protocols described previously (Simonsen et al., 2002; Abdallah et al., 2005). The hMSC-TERT20 cell line was kindly provided by the Molecular and Cell Biology (MCB) Laboratory of the College of Dentistry, in collaboration with the Prince Naif Bin Abdulaziz Health Research Center (King Saud University, Riyadh, Saudi Arabia).

The cell line was cultured in a T-75 culture flask (NEST Scientific, Woodbridge, NJ, USA) with Dulbecco's Modified Eagle's medium (DMEM) with 4 mM glutamine, 1 mM sodium pyruvate, and 4500 mg/L of glucose supplemented with 10% fetal bovine serum (FBS), 1% non-essential amino acids, and 1% antibiotic–antimycotic solution (all Gibco, Invitrogen, Carlsbad, CA, USA) and was maintained at 37°C in a 5% CO_2_ humidified NU-4850E incubator (NuAire^®^ Inc., Plymouth, MN, USA) until ~80% confluence was reached. The medium was replaced every 48 h to maintain stable growth conditions for the cell culture.

### 2.11 Stem cell viability assessment

The viability and proliferation of hBMMSCs was examined by using Alamar Blue™ (Invitrogen™, Eugene, OR, USA) according to the manufacturer's instructions (Cassiano et al., 2022). Each experiment was performed on cells from three independent passages and included two negative controls (untreated cells in basal media and cells treated with 0.2% DMSO). Similar to previous bacterial assessments, BITC concentrations used for all the cell experiments are (0.05% to 0.8%) and Ca(OH)_2_ served as a positive control group. Before use and following UV sterilization, all the test groups were diluted with DMEM immediately before treatment in order to dilute the final concentration of DMSO to 0.2% to eliminate the influence of DMSO. All experimental procedures were performed under aseptic conditions under a Class II laminar flow hood (NU-425-400G Biological Safety Cabinet; NuAire^®^ Inc.).

In brief, the cells were cultured in 96-well flat-bottom culture plates (NEST Scientific, Woodbridge, NJ, USA) using supplemented DMEM at a seeding density of 1×10^4^ cells/well in a volume of 200 μL and allowed to adhere overnight. The culture medium was then removed, and the cells were exposed to 200 µL of experimental and control groups for 1, 2, 4, and 7 days. After the exposure period, the treated medium was removed from the plates, a 1/10^th^ volume of Alamar Blue™ reagent solution was mixed with serum-free DMEM and added to each well, and the plates were incubated in the dark at 37 °C for 4 h. The fluorescence readings of each well were measured at an excitation wavelength of 530 nm and an emission wavelength of 590 nm using a Synergy HT fluorescence microplate reader. Cell viability was calculated using the following formula:


$$\text{Cell viability }\%\;=\;\left(\text{mean FI of cells treated with BITC or Ca}{\left(\text{OH}\right)}_{2/}\text{mean FI of untreated control}\right)\;\times \;100$$

The results are presented as percentages of cell viability in comparison to the control group, and the fluorescence values of the untreated cells were considered to have 100% viability based on ISO 10993–5 guidelines (ISO 10993-5, 2009). The experiment was performed in six wells per condition and repeated three different times.

### 2.12 Cellular morphology assessment

For the observation of any morphological changes, the cells were cultivated (1.5×10^5^ cells/well) on 12-well culture plates (Greiner Bio-One GmbH, Frickenhausen, Germany) and grown until 80% confluency (Ahmed et al., 2016). Subsequently, the cells were treated experimental and control groups. The cellular morphology was observed, and multiple representative fields (in at least 4 replicates) were photographed at different intervals during the 7-day incubation period using an inverted light microscope (Carl Zeiss Axiovert 40C Imaging Microscope, Göttingen, Germany).

### 2.13 Statistical analysis

All data are presented as means ± standard deviations. All the group values were obtained by averaging at least three or more biological replicates. Statistical differences were analyzed using one-way or two-way ANOVA according to the variables analyzed, followed by Tukey's *post hoc* test to test statistical differences among the treatment groups. The statistical analysis was performed using GraphPad Prism (version 6.00 for Windows, La Jolla, CA, USA; https://www.graphpad.com). *p* ≤ 0.05 was considered to be statistically significant.

## 3 Results

### 3.1 Minimum inhibitory concentration and minimum bactericidal concentration

**Figure 1:**
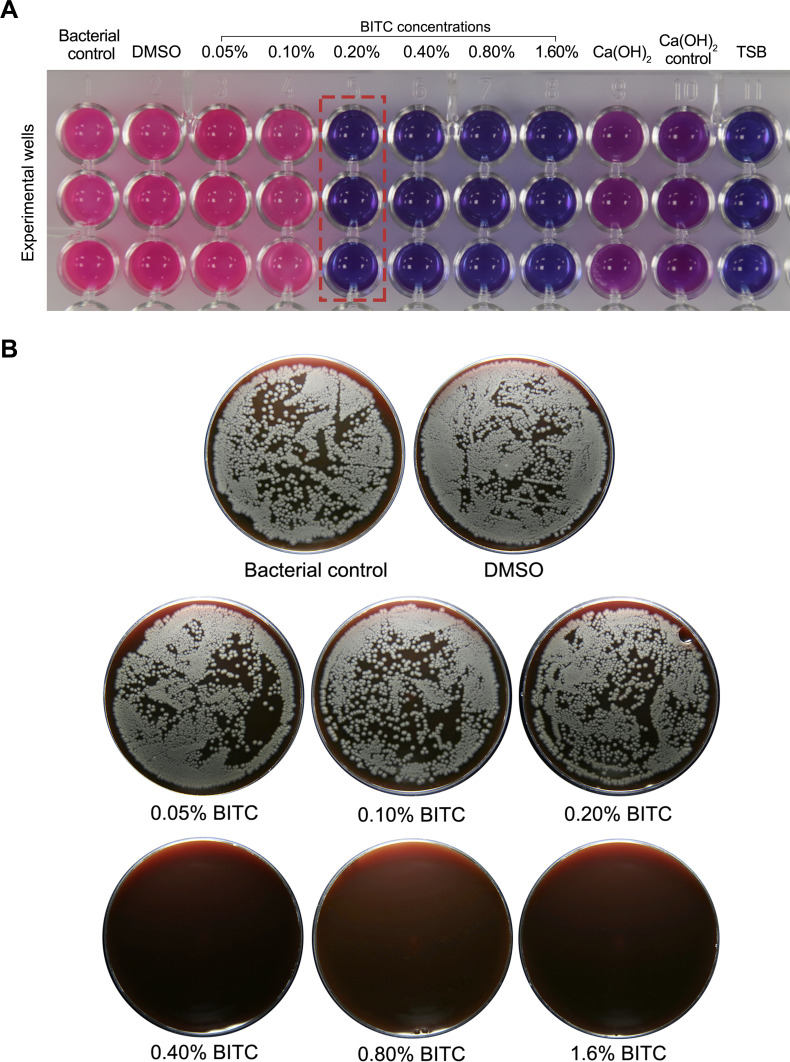
**(A)** Microplate resazurin-based assay showing *F*. *nucleatum* viability. BITC at 0.2% marked the initial indication of bacterial inhibition (highlighted in red), which was determined to be the MIC. **(B)** Agar plate cultures showing visible bacterial colonies in the control and BITC up to 0.2%, while BITC at 0.4% achieved the first visible bacterial elimination, which was identified as the MBC.

BITC at 0.2% exhibited the first observable color shift, indicating bacterial growth inhibition, and was thus identified as the MIC (**Figure 1A**). In agar plate cultures (**Figure 1B**), BITC at 0.4% was identified as the MBC, being the lowest concentration to achieve complete bacterial eradication. Subinhibitory concentrations of ¼ MIC (0.05%), ½ MIC (0.1%), along with MIC (0.2%), MBC (0.4%), and MBC ×2 (0.8%) were selected for further microbiological and cellular analysis.

### 3.2 pH measurements

The pH values of the experimental groups are summarized in **Table 1**. BITC solutions showed slight acidity, exhibiting a minimal decrease in pH over time. In contrast, Ca(OH)_2_ maintained a strongly alkaline pH throughout the incubation period, whereas the TSB control remained near neutral.

**Table 1:** pH values of experimental solutions at days 1, 3, and 7.

Group	pH		
	Day 1	Day 3	Day 7
TSB	7.18 (± 1.08)	7.10 (± 1.44)	7.05 (± 1.79)
¼ MIC	7.08 (± 1.07)	6.99 (± 1.03)	6.82 (± 1.61)
½ MIC	6.87 (± 2.63)	6.77 (± 3.16)	6.73 (± 1.11)
MIC	6.75 (± 2.22)	6.70 (± 1.04)	6.48 (± 1.29)
MBC	6.54 (± 1.12)	6.38 (± 1.54)	6.22 (± 1.08)
MBC ×2	6.37 (± 1.84)	6.24 (± 2.39)	6.19 (± 2.60)
Ca(OH)_2_	12.37 (± 3.66)	12.28 (± 2.08)	11.88 (± 3.48)

### 3.3 Planktonic bacterial viability assessment

**Figure 2:**
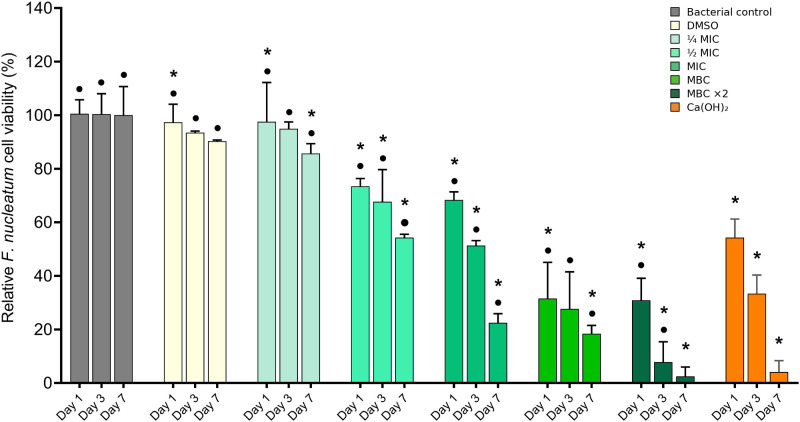
Assessment of planktonic *F. nucleatum* viability using a resazurin-based assay on days 1, 3, and 7. Quantification of the bacterial viability percentage is shown in the bar graph as the mean ± standard deviation (SD). Asterisks (*) indicate *p* ≤ 0.05 compared with the bacterial control at each time point. Dots (•) indicate *p* ≤ 0.05 compared with Ca(OH)_2_ at each time point.

The bacterial control and DMSO groups maintained high bacterial viability at all time points. On day 1, BITC at ¼ MIC, ½ MIC, and MBC significantly reduces viability compared to bacterial control (*p≤*0.05), while the Ca(OH)_2_ group showed greater reduction (*p≤*0.05). In contrast, BITC at MBC and MBC *x*2 showed significantly greater bacterial reduction than the Ca(OH)_2_ group (*p≤*0.05). On day 3, BITC at ½ MIC and MIC remained effective (*p≤*0.05), but was notably lower than MBC, MBC ×2, and Ca(OH)_2_ groups (*p≤*0.05). By day 7, all tested groups demonstrated a significant reduction in bacterial viability when compared to bacterial control (*p≤*0.05). However, MBC ×2 and Ca(OH)_2_ showed the highest antibacterial effects, almost eliminating all *F. nucleatum* planktonic cells, with no statistically significant difference observed (*p* = 0.99) (**Figure 2**).

### 3.4 Reactive oxygen species production

**Figure 3:**
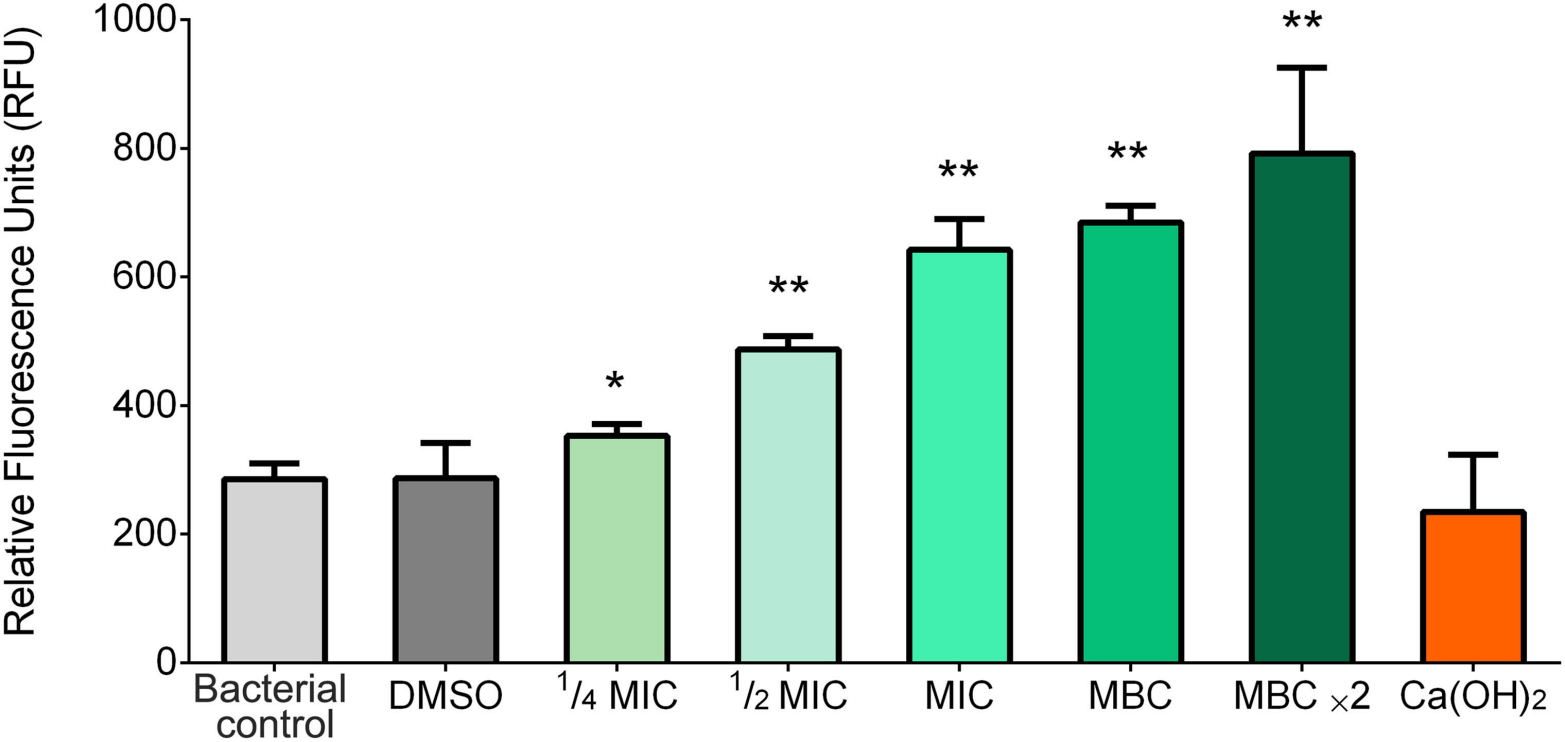
Intercellular ROS levels in *F. nucleatum* following 6 h exposure to experimental groups. Bars represent mean RFU ± SD, (*p* ≤ 0.05) (*) and (*p* ≤ 0.01) (**) compared to the bacterial control group.

BITC exposure led to increased ROS production in *F. nucleatum*, as reflected by elevated fluorescence levels across all treated groups (**Figure 3**). The highest ROS activity was observed in the MBC ×2 group, suggesting greater oxidative stress with higher concentrations. However, with Ca(OH)_2_ treatment, no significant change in ROS levels relative to the control was noted.

### 3.5 Biofilm biomass quantification

**Figure 4:**
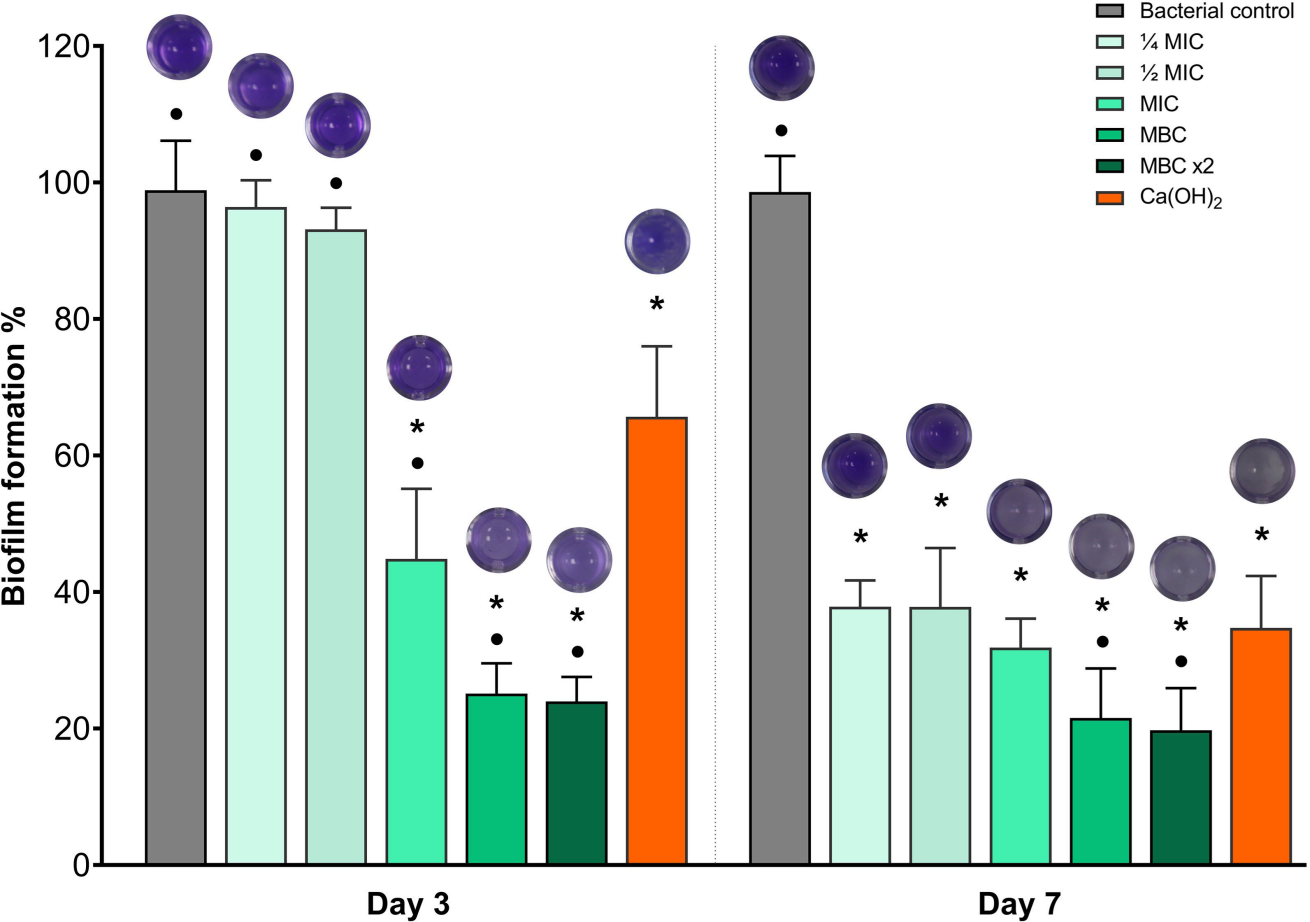
Crystal violet assay showing biofilm formation (%) on days 3 and 7 following treatment. Bars represent the mean ± SD of biofilm formation. The stained wells are displayed above each bar. Asterisks (*) indicate *p* ≤ 0.05 compared with the bacterial control at each time point. Dots (•) indicate *p* ≤ 0.05 compared with Ca(OH)_2_ at each time point.

Biofilm biomass formation was quantified using the crystal violet biofilm assay (**Figure 4**). On day 3, BITC at ¼ MIC and ½ MIC exhibited minimal biofilm reduction with no significant difference in comparison to the bacterial control group (*p* >0.05). The Ca(OH)_2_ group showed a significant reduction in biofilm formation compared with the bacterial control group (*p* ≤ 0.05). However, the BITC at MIC, MBC, and MBC ×2 demonstrated a more significant reduction compared to the Ca(OH)_2_ group (*p* ≤ 0.05). By day 7, all experimental groups showed significant biofilm reduction compared to the bacterial control group (*p* ≤ 0.05). The BITC at ≤ MIC showed no significant difference in biofilm reduction compared with the Ca(OH)_2_ group (*p* >0.05). However, BITC at MBC and MBC ×2 exhibited significantly greater biofilm reduction than the Ca(OH)_2_ group (*p* ≤ 0.05).

### 3.6 Biofilm viability assessment

**Figure 5:**
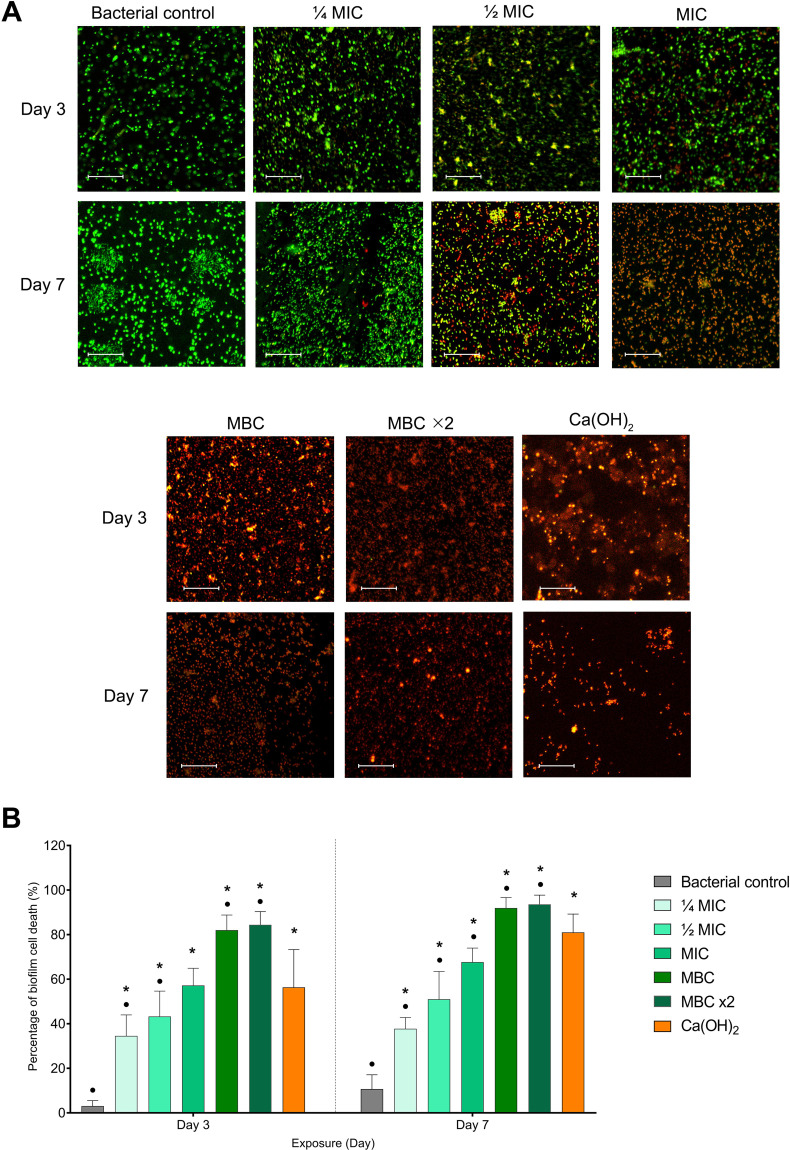
**(A)** Representative fluorescence images of *F*. *nucleatum* biofilm cells stained for viability (green=live, red=dead). **(B)** Quantification of bacterial cell death percentage is shown in the bar graph as means ± SD. Asterisks (*) indicate *p* ≤ 0.05 compared with the bacterial control at each time point. Dots (•) indicate *p* ≤ 0.05 compared with Ca(OH)_2_ at each time point.

Live/dead fluorescent imaging revealed a significant increase in biofilm cell death in all experimental groups compared to the untreated bacterial control at both time points (*p* ≤ 0.05). CLSM images demonstrated strong green fluorescence intensity in the bacterial control group at both time points, indicating a high proportion of live bacteria. In contrast, experimental groups showed an increased red fluorescence supporting bactericidal efficacy (**Figure 5A**). On day 3, BITC at ¼ MIC and ½ MIC demonstrated significantly less biofilm cell death compared to the Ca(OH)_2_ group (*p* ≤ 0.05), while no significant difference was observed between the MIC and Ca(OH)_2_ groups (*p* >0.05). However, BITC at MBC and MBC ×2 led to significantly greater bactericidal effects than the Ca(OH)_2_ group (*p* ≤ 0.05). By day 7, BITC at ≤ MIC were significantly less effective than the Ca(OH)_2_ group (*p* ≤ 0.05). On the contrary, MBC and MBC ×2 exhibited significantly higher levels of biofilm cell death than the Ca(OH)_2_ group (*p* ≤ 0.05) (**Figure 5B**).

### 3.7 Biofilm morphology analysis

**Figure 6:**
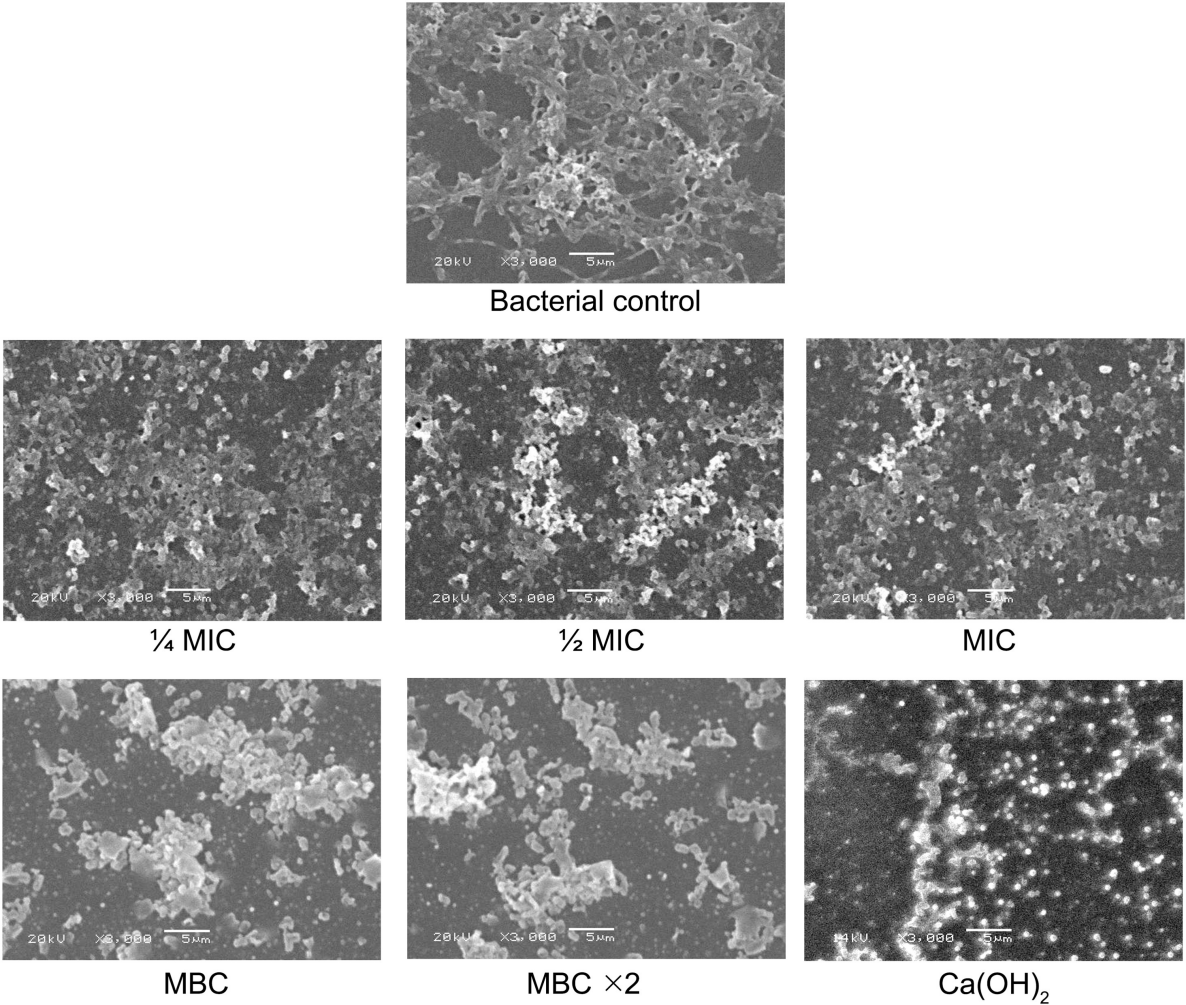
SEM images of *F. nucleatum* biofilm morphology after exposure to experimental groups. Scale bar = 5 μm.

SEM analysis of biofilm morphology demonstrated notable structural differences across examined groups (**Figure 6**). The bacterial control group exhibited a dense, well-organized biofilm with abundant extracellular matrix. In BITC at ¼ MIC and ½ MIC groups, bacterial clusters remained visible with slight disruption noted. However, MIC and MBC led to noticeable structural damage, with deformed cells and extracellular matrix breakdown. Notably, treatment with MBC ×2 and Ca(OH)_2_ caused extensive biofilm disruption, scattered cellular debris, and loss of structural integrity.

### 3.8 Stem cell viability assessment

**Figure 7:**
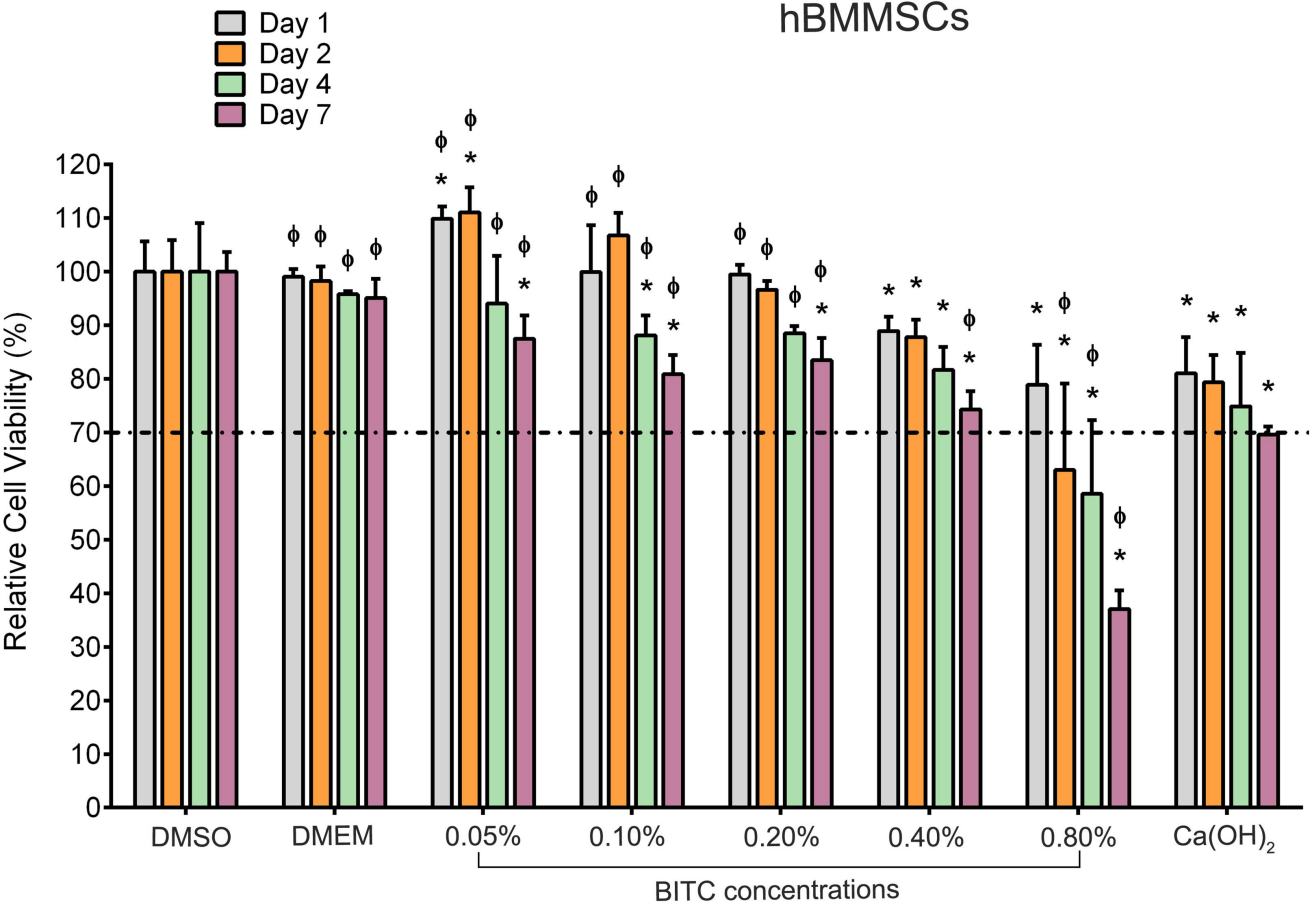
hBMMSCs following exposure to experimental groups over a 7-day time period using the Alamar Blue™ assay. According to the ISO 10993–5 guidelines, the cell viability threshold was set at 70% (dashed line). Bars represent the mean ± SD of cell viability. Asterisks (*) indicate *p* ≤ 0.05 compared with DMEM control at each time point. The phi symbol (Φ) indicates *p* ≤ 0.05 compared with Ca(OH)_2_ at each time point.

Alamar Blue™ viability assay was used to evaluate the effects of experimental groups on the viability of hBMMSCs. The results are presented as percentages of cell viability relative to the untreated control group (**Figure 7**). According to ISO 10993-5:2009 guidelines for the biological evaluation of medical devices, a reduction in viability below 70% is considered indicative of cytotoxicity (ISO 10993-5, 2009).

The DMSO-treated stem cells exhibited minimal reductions in viability, suggesting that DMSO, as a vehicle, exerted a negligible effect on cell survival. In the BITC 0.05% and 0.1% groups, an initial proliferative response was noted at days 1 and 2, respectively. This increase was statistically significant only in the 0.05% group compared to the negative control group (DMEM). Over time, both groups exhibited a gradual decline in proliferation, with significant reductions observed at day 7. In contrast, higher concentrations (0.2% and 0.4%) caused a decrease in cell counts over the exposure period. For the 0.2% group, the decline became significant by days 4 and 7, while the 0.4% group showed a significant reduction at all time points. Despite these trends, cell viability remained relatively stable in the previously mentioned groups over the 7-day period. However, the BITC 0.8% group displayed a clear decline in cell viability starting as early as day 2, with cell counts continuing to decline significantly over time. Similarly, cells exposed to Ca(OH)_2_ also experienced a significant drop in viability across the exposure period. When comparing treatment groups, cells exposed to the Ca(OH)_2_ had significantly lower cell counts than the BITC 0.05%, 0.1%, and 0.2% groups at all time points (*p ≤* 0.05). No significant difference was found between Ca(OH)_2_ and BITC 0.4% (*p >*0.05), while BITC 0.8% induced significantly greater cytotoxicity than the Ca(OH)_2_ group (*p* ≤ 0.05).

### 3.9 Cellular morphology assessment

**Figure 8:**
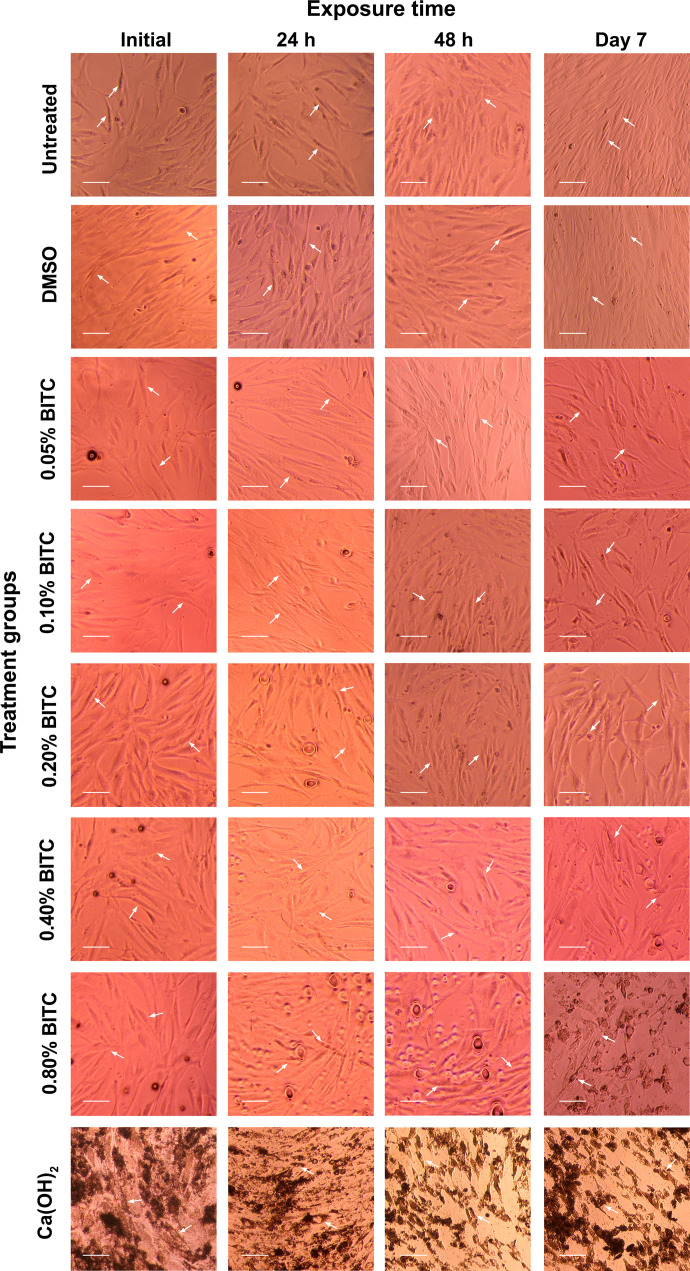
Representative phase-contrast microscopic images showing the morphology of hBMMSCs at the initial stage, 24 h, 48 h, and day 7 in untreated control and experimental treatment groups. White arrows indicate characteristic cell morphology. All images were captured at 10× magnification. Scale bar = 50 µm.

The hBMMSCs morphology was monitored microscopically at baseline, 24 h, 48 h, and day 7 following exposure to experimental groups (**Figure 8**). The control group and DMSO-treated cells maintained a normal spindle-shaped appearance with consistent confluency and proliferation throughout the observation period. BITC at concentrations of 0.05%, 0.1%, 0.2%, and 0.4% maintained a normal confluent spindle-shaped appearance for the first 48 h, with a slight reduction in confluency by day 7. However, the 0.8% BITC group showed evident morphological changes, including cell shrinkage and detachment. Similarly, Ca(OH)_2_ demonstrated dark granulation regions and changes in cell morphology at the 24 h mark, with substantial cellular detachment and loss of confluency by day 7.

## 4 Discussion

In REPs, chemical disinfection is critical not only for eradicating pathogens but also for creating an environment that supports stem cell survival and conduction (Diogenes and Hargreaves, 2017). Improper disinfection of bacterial biofilms can compromise the differentiation potential of stem cells during treatment (Vishwanat et al., 2017). Therefore, the intracanal medicaments used in REPs must strike a balance between potent antibacterial activity and minimal cytotoxicity towards stem cells (Murray et al., 2007).

Although endodontic infections are polymicrobial in nature (Siqueira and Rôças, 2022), *F. nucleatum* was selected in this study due to its high prevalence in root canals before and after chemo-mechanical preparation, and is one of the most frequently detected bacteria in immature permanent teeth with necrotic pulps (Zandi et al., 2018; Fouad, 2019). The antibacterial evaluation revealed that BITC exhibits potent inhibitory activity against *F. nucleatum*, with an MIC of 0.2% and an MBC of 0.4% which is higher than the MIC values reported for other bacterial species in previous studies (Song et al., 2019; Alhandal et al., 2022; Vrca et al., 2023). This might be due to its anaerobic nature, its ability to form a robust dense biofilm, and the upregulation of specific genes that might enhance biofilm resistance (Zhao et al., 2022; Ciani et al., 2024). Notably, BITC effectively eliminated *F. nucleatum* planktonic cells within the first 24 h and disrupted established biofilms by 48 h at MBC concentrations. Moreover, BITC was observed to have a concentration-dependent antibacterial effect on *F. nucleatum* biofilms, with significant reductions in viability observed at 0.4% and 0.8% concentrations. This finding aligns with previous studies reporting similar dose-dependent activity against *Staphylococcus aureus* and *Salmonella typhimurium* (Wang et al., 2019; Niu et al., 2020).

The mechanism of action of BITC is believed to be multifactorial, involving the disruption of cell membranes, suppression of virulence factors, oxidative stress induction, bacterial morphology alteration, and impairment of biofilm adhesion capabilities (Kaiser et al., 2017; Liu et al., 2021; Hoch et al., 2024). In our study, SEM analysis demonstrated an alteration in *F. nucleatum* morphology and biofilm detachment. Crystal violet staining further confirmed a significant reduction in biofilm biomass following treatment, particularly at concentrations corresponding to the MIC and higher. In addition, BITC treatment led to a marked increase of intracellular ROS in *F. nucleatum*, supporting previously reported evidence of oxidative stress induction.

To assess clinical relevance, BITC was compared to calcium hydroxide (CaOH_2_), in the form of UltraCal XS (Ultradent, South Jordan, UT, USA), in accordance with previous *in vitro* and ex vivo regenerative endodontic studies (Althumairy et al., 2014; Latham et al., 2016; Bilvinaite et al., 2024). Our results demonstrated that BITC concentrations corresponding to MIC and higher exhibited a rapid antibacterial effect, resulting in a high proportion of dead bacterial cells and a marked reduction in biofilm formation at early experimental stages. In contrast, Ca(OH)_2_ showed minimal antibacterial effects during the first 72 h. However, by day 7, Ca(OH)_2_ exerted a potent effect, increasing bacterial cell death and reducing biofilm formation. Although this delayed effect was notable, its efficacy remained significantly lower than 0.4% and 0.8% BITC concentrations. This delayed antibacterial action was supported earlier by Sjögren et al., who reported that Ca(OH)_2_ requires at least 7 days for optimal bacterial elimination (Sjögren et al., 1991). The antimicrobial action of Ca(OH)_2_ is mainly attributed to its ability to release hydroxyl ions creating an alkaline environment capable of disrupting bacterial cell membranes (Mohammadi and Dummer, 2011). In the present study, pH evaluation showed that Ca(OH)_2_ maintained high alkalinity throughout the 7-day period.

In addition to the importance of proper microbial disinfection, an antimicrobial agent must also preserve stem cell viability for successful regeneration (Parchami et al., 2024). Various types of mesenchymal stem cells have been identified in REPs, including dental pulp stem cells (DPSCs), apical papilla stem cells (SCAP), periodontal ligament stem cells (PDLSCs), alveolar-bone-derived mesenchymal stem cells (ABSMCs), stem cells from human exfoliated deciduous teeth (SHEDs), and human bone marrow mesenchymal stem cells (hBBMSCs) (Huang et al., 2009; Ayoub et al., 2020). Moreover, studies have validated the use of immortalized human bone marrow mesenchymal stem cells in regenerative research (AlMobarak et al., 2025). In our study, immortalized hBMMSCs was selected to evaluate the viability responses to the experimental materials. The BITC concentrations (*≤*0.4%) maintained stem cell viability. In fact, low BITC concentrations (0.05% and 0.1%) enhanced cell proliferation, increasing the cell count to a greater extent than that for the control group. In contrast, 0.8% BITC showed cytotoxic effects after 48 h and continued to reduce cell counts throughout the observation period. Consistent with the viability findings, morphological assessment showed that BITC groups at (*≤*0.2%), maintained normal spindle-shaped cellular morphology with firm attachment. However, 0.4% BITC exhibited mild alterations such as reduced cellular spreading. Although the current evidence on the effects of BITC on stem cell viability remains limited, findings from structurally related compounds such as moringin suggest positive regeneration potential. In hPDLSCs, moringin increases stem cell proliferation and promotes neural differentiation, while in human gingival mesenchymal stem cells (GMSCs), it maintains cell viability and promotes regenerative responses (Romeo et al., 2018a; Chiricosta et al., 2019). Moreover, isothiocyanates as a class demonstrated a similar behavior, where low concentrations protected mesenchymal stem cells (MSCs) from oxidative injuries, while higher concentrations led to DNA damage and cytotoxicity (Zanichelli et al., 2012), supporting the dose-dependent effect observed in our study.

On the other hand, Ca(OH)_2_ demonstrated a significantly higher cytotoxicity towards hBMMSCs, with cell viability initially maintained but declined as exposure progressed. Morphological analysis confirmed this unfavorable effect by showing evident signs of cell stress, including rounding, detachment, and shrinkage. Comparable findings have been reported previously, in which Ca(OH)_2_ significantly reduced the viability and proliferation of bone marrow-derived mesenchymal stem cells (Khosropanah et al., 2018). The cytocompatibility of BITC compared to Ca(OH)_2_ suggests that BITC is a potential candidate for REPs, where preserving stem cell function is critical. Furthermore, its natural origin and known safety profile add to its translational appeal for clinical application.

This study has several limitations, which should be acknowledged. First, the antibacterial evaluation and cell viability assessments were limited to *in vitro* models, which do not fully represent the complex biological reactions present in biological systems. Secondly, the antibacterial evaluation was only conducted on mono-species *F. nucleatum*; although one of the most prevalent bacteria in endodontic infections, it does not represent the diverse microbial species detected in clinical settings (Siqueira and Rôças, 2022). Lastly, the stem cell assessment was limited to viability and morphology effects over a 7-day observation period. It did not evaluate the long-term effects on stem cell function, including differentiation potential, migration, mineralization capabilities, and gene expression. Future studies should also explore the efficacy of BITC in multispecies biofilm models, co-culture systems, and *in vivo* models. In addition, exploring controlled delivery systems or sustained-release formulations for intracanal use may optimize BITC's clinical utility.

## 5 Conclusions

In conclusion, benzyl isothiocyanate demonstrated promising antibacterial and antibiofilm activity against *Fusobacterium nucleatum* compared to calcium hydroxide. Moreover, benzyl isothiocyanate preserved the viability and morphology of human bone marrow mesenchymal stem cells, whereas calcium hydroxide showed cytotoxic effects and caused notable morphological alterations. These preliminary findings suggest that benzyl isothiocyanate may serve as a potential bioactive antimicrobial agent relevant for regenerative endodontic applications. However, further research is needed to evaluate its efficacy against a broader range of pathogens and to optimize concentrations that balance intracanal disinfection with biological compatibility.

## Data Availability

The raw data supporting the conclusions of this article will be made available by the authors, without undue reservation.

## Glossary

ABSMCs: Alveolar bone-derived mesenchymal stem cells
BITC: Benzyl isothiocyanate
Ca(OH)_2_: Calcium hydroxide
CLSM: Confocal laser scanning microscopy
DMEM: Dulbecco's Modified Eagle's medium
DMSO: Dimethyl sulfoxide
DPSCs: Dental pulp stem cells
*F. nucleatum*: *Fusobacterium nucleatum*
hBMMSCs: Human bone marrow mesenchymal stem cells
hGMSCs: Human gingival mesenchymal stem cells
hPDLSCs: Human periodontal ligament stem cells
MIC: Minimum inhibitory concentration
MBC: Minimum bactericidal concentration
REPs: Regenerative endodontic procedures
ROS: Reactive oxygen species
SCAP: Stem cells of apical papilla
SEM: Scanning electron microscopy
SHEDs: Stem cells from human exfoliated deciduous teeth
TSA: Tryptic soy agar
TSB: Tryptic soy broth
