# Supplementary material for: Benzyl isothiocyanate exhibits antibacterial and antibiofilm activity against Fusobacterium nucleatum while preserving viability of human bone marrow mesenchymal stem cells: an in vitro study
Source: Front Cell Infect Microbiol. 2025 Oct 24;15:1683203. doi: 10.3389/fcimb.2025.1683203 (PMC12592193; doi:10.3389/fcimb.2025.1683203)
Supplement: Supplementary file 1 [file Table1.docx]

**Table 1.** Mean *F. nucleatum* biofilm death percentage and statistical comparisons

| **Group** | **Day 3** | | | **Day 7** | | | |
| --- | --- | --- | --- | --- | --- | --- | --- |
|  | Mean cell death % (± SD) | P value vs. control | P value vs. UltraCal XS | Mean cell death % (± SD) | P value vs. control | P value vs. UltraCal XS | |
| Control | 3.06% (±2.47) | NA | *p* ≤ 0.05 | 10.70% (±6.43) | NA | | *p* ≤ 0.05 |
| BITC05 | 34.56% (±9.43) | *p* ≤ 0.05 | *p* ≤ 0.05 | 37.75% (±5.07) | *p* ≤ 0.05 | | *p* ≤ 0.05 |
| BITC1 | 43.28% (±11.40) | *p* ≤ 0.05 | *p* ≤ 0.05 | 51.02% (±12.48) | *p* ≤ 0.05 | | *p* ≤ 0.05 |
| BITC2 | 57.19% (±7.75) | *p* ≤ 0.05 | *p*$=$0.99 | 67.65% (±6.34) | *p* ≤ 0.05 | | *p* ≤ 0.05 |
| BITC4 | 82.04% (±6.78) | *p* ≤ 0.05 | *p* ≤ 0.05 | 91.94% (±4.73) | *p* ≤ 0.05 | | *p* ≤ 0.05 |
| BITC8 | 84.43% (±5.93) | *p* ≤ 0.05 | *p* ≤ 0.05 | 93.61% (±4.16) | *p* ≤ 0.05 | | *p* ≤ 0.05 |
| UltraCal XS | 56.33% (±16.98) | *p* ≤ 0.05 | NA | 81.01% (±8.22) | *p* ≤ 0.05 | | NA |

**Table 2.** Mean F. nucleatum biofilm formation percentage and statistical comparisons

| **Group** | **Day 3** | | | **Day 7** | | | |
| --- | --- | --- | --- | --- | --- | --- | --- |
|  | Biofilm Formation % (± SD) | P value vs. control | P value vs. UltraCal XS | Biofilm Formation % (± SD) | P value vs. control | | P value vs. UltraCal XS |
| Control | 100% (±7.29) | *NA* | *p* ≤ 0.05 | 100% (±5.30) | *NA* | *p* ≤ 0.05 | |
| BITC05 | 96.42% (±3.92) | *p = 0.99* | *p* ≤ 0.05 | 37.85% (±3.88) | *p* ≤ 0.05 | *p = 0.97* | |
| BITC1 | 93.14% (±3.16) | *p = 0.52* | *p* ≤ 0.05 | 37.83% (±8.64) | *p* ≤ 0.05 | *p = 0.97* | |
| BITC2 | 44.85% (±10.29) | *p* ≤ 0.05 | *p* ≤ 0.05 | 31.87% (±4.25) | *p* ≤ 0.05 | *p = 0.98* | |
| BITC4 | 25.12% (±4.45) | *p* ≤ 0.05 | *p* ≤ 0.05 | 21.54% (±7.27) | *p* ≤ 0.05 | *p* ≤ 0.05 | |
| BITC8 | 23.96% (±3.60) | *p* ≤ 0.05 | *p* ≤ 0.05 | 19.76% (±6.18) | *p* ≤ 0.05 | *p* ≤ 0.05 | |
| UltraCal XS | 65.67% (±10.35) | *p* ≤ 0.05 | *NA* | 34.76% (±7.62) | *p* ≤ 0.05 | *NA* | |
